# The effect on dental care utilization from transitioning pediatric Medicaid beneficiaries to managed care

**DOI:** 10.1002/hec.4496

**Published:** 2022-03-23

**Authors:** Kamyar Nasseh, John R. Bowblis

**Affiliations:** ^1^ Health Policy Institute American Dental Association Chicago Illinois USA; ^2^ Department of Economics Miami University Oxford Ohio USA

**Keywords:** dental care, fee‐for‐service, managed care, Medicaid, quasi‐experimental research methods

## Abstract

Compared to the fee‐for‐service (FFS) model, the managed care delivery system has the potential to improve health care management, increase provider accountability, and support better monitoring of health care quality. However, managed care organizations may attempt to control costs by curbing utilization among Medicaid beneficiaries or reducing reimbursement for Medicaid services. It is an empirical question whether managed care increases or decreases utilization of services. Using detailed pediatric public insurance dental claims data from 2016 through 2018, we examined whether the transition from FFS to managed care affects rates of dental care utilization. Between 2016 and 2018, Indiana, Missouri and Nebraska transitioned pediatric Medicaid beneficiaries from public dental fee‐for‐service programs to private managed care entities. Using an extended two‐way fixed‐effects estimation framework, we found that dental managed care leads to a decline in dental care utilization, especially when compared to states that maintain FFS provision of Medicaid dental services.

## 1 INTRODUCTION

Historically, states have used fee‐for‐service (FFS) delivery models for their Medicaid programs. Under FFS, providers are reimbursed for each billable service, providing no incentive for providers to contain cost, use the most cost‐effective treatments, or provide services that are not reimbursed. These ever‐increasing health care costs put a strain on public finances, particularly at the state level where Medicaid accounts for about 29% of total state spending (Rudowitz et al., 2021). In recent years, states have attempted to control Medicaid costs by switching to private managed care organizations (MCOs) to deliver Medicaid services, with over four out of every five Medicaid beneficiaries enrolled in some form of managed care in 2018 (Medicaid.gov, 2018a). As of July 2019, 40 states including the District of Columbia contract with MCOs to provide comprehensive risk‐based health care plans for at least some Medicaid beneficiaries (Hinton et al., 2020).

Advocates for managed care argue that when compared to FFS, private MCOs have greater expertize and resources and are better able to manage the health of Medicaid beneficiaries through pay‐for‐performance incentives. This means MCOs potentially improve health care management, increase provider accountability, and support better monitoring of health care quality while offering states greater control and predictability about future costs (MACPAC, n.d. (a)). At the same time, MCOs may reduce utilization and expenditures by restricting the number of in‐network providers and lowering reimbursement rates paid to those providers. Additionally, some arrangements pay MCOs capitated rates per beneficiary, further incentivizing MCOs to control costs (MACPAC, n.d. (b)).

While there is a general move to MCOs to cover traditional health care services, many states continue to cover specialized services such as long‐term care, behavioral health, and dental services under traditional FFS Medicaid. There is little evidence comparing MCOs to traditional FFS plans in Medicaid programs when it comes to these specialized services. There are several studies examining access to and demand for Medicaid dental services after states either increased provider reimbursement or expanded Medicaid dental benefits (Buchmueller et al., 2015; 2016; Choi, 2011; Decker, 2011). However, these studies did not examine dental care utilization when Medicaid programs transition dental benefits from FFS to managed care. In this paper, we address this gap by focusing on the role of MCOs in delivering dental services. There are a number of studies that examine the role of managed care in Medicaid dental services (Burns, 2009; Coughlin & Long, 2000; Marton et al., 2012; Zuckerman et al., 2002). However, these studies lacked repeated cross‐sectional data before and after the transition to managed care or lacked a comparison group of Medicaid beneficiaries in states that stay under FFS Medicaid for dental services.

It is important for policymakers to focus on pediatric dental services because access to dental care, particularly at a young age, can affect development and productivity in future years. Good oral health in childhood can lead to better labor market outcomes later in life (Glied & Neidell, 2010). Fortunately, dental care use among children has increased since the early 2000s and is at or near its highest recorded level (American Dental Association, 2017a). This increase in pediatric dental care utilization has been driven primarily by publicly insured children (American Dental Association, 2017a; Crall & Vujicic, 2020). Also, racial disparities in untreated caries (e.g., cavities) is narrowing among children (American Dental Association, 2017b). Hence, it is important for policymakers to know if private provision of pediatric Medicaid dental services through MCOs could enhance or reverse the progress children have made over the last 20 years with respect to utilization of dental services. That is an open question we hope to answer in this paper.

Furthermore, the role MCOs play in providing dental services is not without controversy. For example, Maryland implemented managed care for pediatric dental services in 1997 in an effort to improve dental service quality, but the MCOs did not increase dentist participation or utilization among Medicaid beneficiaries (Thuku et al., 2012). After the death of a pediatric Medicaid dental patient in 2007, Maryland carved its Medicaid dental program out of managed care in 2009. Reimbursement rates were increased and administrative services were streamlined through a single vendor (Thuku et al., 2012).

In this study, we utilized difference‐in‐differences estimation to measure how dental care utilization among pediatric Medicaid beneficiaries changed in three states (Indiana, Missouri and Nebraska) that transitioned from FFS to managed care between 2016 and 2018 relative to 18 states that maintained universal FFS provision of Medicaid dental services over the same time period. We relied on dental claims data from the Transformed Medicaid Statistical Information System (T‐MSIS). The pediatric population is studied because all states are mandated to cover dental benefits for children under age 21 in Medicaid^1^ through the Early and Periodic Screening, Diagnostic and Treatment (EPSDT) benefit. As of 2016, 21 states^2^ and the District of Columbia had pediatric Medicaid dental benefits administered by MCOs (Gupta et al., 2017). In contrast, Medicaid dental coverage for adults is optional, and adult dental benefits vary across states (Medicaid.gov, n.d.). Among the limited number of states that also provided adult dental benefits, five states had their dental program administered by MCOs (Gupta et al., 2017). Therefore, by studying the pediatric population, we examine the role of MCOs in a specialized service area where dental benefits are comprehensive and there is less heterogeneity across states.

Our findings indicate that dental care utilization, measured as visits per 10,000 beneficiaries and share of beneficiaries with a dental claim, declined following adoption of dental managed care, especially in the first few quarters after implementation. Utilization in Indiana and Nebraska also decreased across dental service categories (diagnostic and preventive) and for specific procedures (prophylaxis and fluoridation). There was weaker evidence that utilization for restorative dental services declined significantly in the three states following the transition to dental managed care.

The paper is structured as follows: Section 2 provides a conceptual framework for our analysis and a timeline of the managed care reforms in Indiana, Missouri and Nebraska; Section 3 describes the dental claims data; Section 4 provides an overview of our empirical strategy; Section 5 presents the results; and Section 6 concludes the paper, exploring the health policy implications of our findings.

## 2 CONCEPTUAL FRAMEWORK AND TIMELINE

In an effort to limit health care costs and improve outcomes for patients, public payers such as Medicaid and Medicare have transitioned from the public provision of health care services via traditional FFS models to private provision of services through MCOs. Proponents of MCOs suggest that private provision of public services leads to greater patient outreach, better case management, improved administrative services for providers, and improved health outcomes. These characteristics could increase utilization. At the same time, MCOs can use their size and financial incentives to channel patients to preferred in‐network providers who accept lower prices (Wu, 2009). Managed care organizations are often reimbursed under risk‐based contracts with capitated arrangements, meaning that MCOs have an incentive to keep expenditures below a certain threshold.

This may be one reason why the existing literature finds mixed results in terms of health care outcomes, utilization, expenditures and consumer welfare when states switch from publicly provided FFS to a private managed care organizations (MCO) model (Aizner et al., 2007; Curto et al., 2019; Dranove et al., 2021; Duggan, 2004; Duggan & Morgan, 2010; Town & Liu, 2003). The ultimate effect depends in part on the level of spending and provider reimbursement in the FFS program prior to the transition to managed care (Duggan & Hayford, 2013). For example, when FFS programs are heavily rationed, private provision of health care services within public programs could lead to better health outcomes; conversely, when FFS programs are more generous, a transition to managed care could lead to worse health outcomes (Layton et al., 2019).

One would expect that the mechanisms through which managed care affects health care utilization to be the same as the mechanisms through which managed care affects specialized care, such as dental services. However, there may be some key differences. In particular, managed care entities handling health care services outside of dental care may use care coordination across various health care services because these health care services could be interlinked or the same service could be provided in multiple settings. For example, physical therapy after a hospitalization could be linked and provided at the patient's home, an outpatient rehab center, or a skilled nursing facility. However, the provision of dental services is often siloed from other health care services and are generally provided in one setting. Hence, one may expect the impact of managed care on dental service utilization to be different from the impact of managed care provision on medical services.

Within the context of pediatric dental services, states are mandated to cover basic services, but the relative budget the state allocates to dental services under FFS and after transitioning to managed care may affect the utilization of dental services. Managed care organizations may be able to utilize their expertize to enhance access to dentists. However, MCOs must still operate within the budgets provided to them by the state. Therefore, less generous payments to the MCO will require it to engage in strategies to provide services within the budget they are provided. This means MCOs may negotiate with dentists to pay lower rates, which could ultimately lead to some dentists being excluded from an MCO's network. Furthermore, MCOs may cap basic care (e.g., X‐rays, prophylaxis, sealants) to a certain number of services per year, which could lower utilization among Medicaid beneficiaries. Overall, it is an empirical question whether the transition of dental services from FFS to managed care increases or decreases dental care utilization.

**FIGURE 1:**
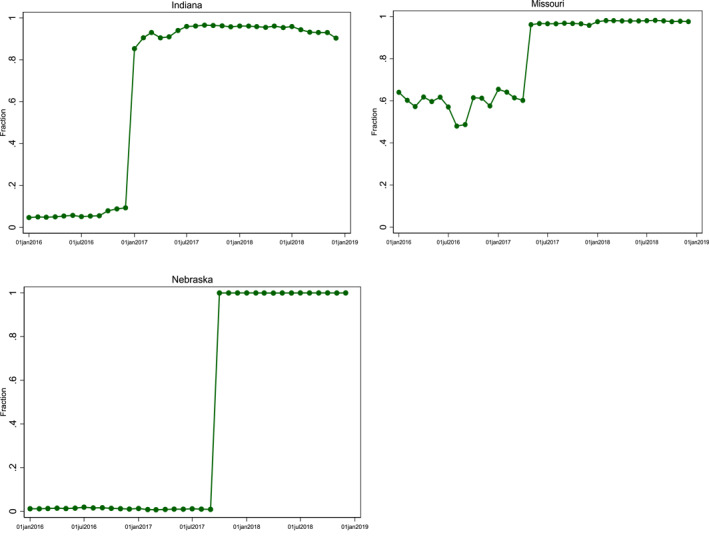
Fraction of pediatric medicaid/CHIP dental claims in Indiana, Missouri and Nebraska paid by managed care (2016–2018). Fraction of claims that are managed care in Missouri take into account data from all counties in the state. However, all subsequent analysis only considers counties in Missouri that transitioned to dental managed care in May 2017. Counties in Missouri that transitioned to dental managed care prior to May 2017 are dropped from all regression analysis. Dates states transitioned to managed care are: Jan. 1, 2017 (Indiana); May 1, 2017 (Missouri); Oct. 1, 2017 (Nebraska). *Source*: 2016–2018 T‐MSIS Medicaid and CHIP Analytic Claims Data for children ages 0–20

In this study, we examined three states that transitioned their Medicaid pediatric dental benefits from FFS to managed care. Indiana, Missouri and Nebraska made this transition at different points in 2017.^3^ Table 1 provides a summary of the managed care programs in these three states. Indiana implemented their Medicaid managed care program, Hoosier Health wise (HHW), in 1997 but continued to provide pediatric dental services on a FFS basis (Medicaid.gov, 2014). On January 1, 2017, Indiana mandated all dental services for children under the age of 19 be administered via HHW (In.gov, n.d.; Medicaid.gov, 2017a). Four MCOs administered pediatric Medicaid dental benefits in Indiana: DentaQuest on behalf of Anthem, CareSource, MDWise, and Managed Health Services (DentaQuest, undated; In.gov, undated). The transition to managed care occurred quickly, with only 9% of pediatric dental claims associated with MCOs in December 2016, rising to over 85% in January 2017, and then to 90%–96% thereafter (Figure 1).

**TABLE 1:** Summary of pediatric dental managed care in Indiana, Missouri and Nebraska

	Indiana	Missouri	Nebraska
Date of transition from FFS to dental managed care	January 01, 2017	January 05, 2017	January 10, 2017
Type of MCO contract	Comprehensive (medical and dental)	Comprehensive (medical and dental)	Pre‐paid ambulatory health plan (dental carve‐out)
Regions effected	Statewide	Southwest and Central Missouri. Prior to January 5, 2017 the counties around St. Louis, Kansas City and Columbia, Missouri transitioned to managed care	Statewide
Populations enrolled	All children (disabled and non‐disabled) except those in foster care or receiving adoptive assistance	All children (disabled and non‐disabled) except those in foster care or receiving adoptive assistance	All children (disabled and non‐disabled).
Dental services covered	Preventive, corrective, medical and surgical	Preventive, corrective, medical and surgical	Preventive, corrective, medical and surgical
Number of participating plans	4	12	1
Are dental services carved out from other medical services?	No	No	Yes
Type of payment	Capitated payment covers dental and other services	Capitated payment covers dental and other services	Dental‐specific capitated payment

Abbreviations: FFS, fee‐for‐service; MCO, Managed care organization; PAHP, Prepaid ambulatory health plan.

*Source*: MACPAC, n.d. (b). Types of managed care arrangements. Retrieved from: https://www.macpac.gov/subtopic/types‐of‐managed‐care‐arrangements/. Medicaid.gov (2017a). Indiana managed care program features, as of 2017. Retrieved from: https://www.medicaid.gov/Medicaid/downloads/in‐2017‐mmcdcs.pdf. Medicaid.gov (2017b). Missouri managed care program features, as of 2017. Retrieved from: https://www.medicaid.gov/Medicaid/downloads/mo‐2017‐mmcdcs.pdf/. Medicaid.gov (2017c). Nebraska managed care program features, as of 2017. Retrieved from: https://www.medicaid.gov/Medicaid/downloads/ne‐2017‐mmcdcs.pdf/. Missouri Health Net (2020). Dental manual. Retrieved from: http://manuals.momed.com/collections/collection_den/print.pdf. Nebraska Department of Health and Human Services (2021). Title 482 Managed Care. Chapter 5 Dental Benefits Package. Retrieved from: https://www.nebraska.gov/rules‐and‐regs/regsearch/Rules/Health_and_Human_Services_System/Title‐482/Chapter‐5.pdf.

Missouri staggered its implementation of managed care by region. Medicaid beneficiaries in eastern and western Missouri (e.g., St. Louis and Kansas City regions) began to be covered by MCOs in the mid‐1990s. Southwest Missouri and portions of Central Missouri transitioned their pediatric Medicaid populations to MCOs on May 1, 2017 (Missouri Health Net, 2020). Between January 2016 and April 2017, the percentage of total pediatric Medicaid claims in Missouri paid by MCOs ranged from 48% to 65%. After Missouri implemented managed care in the rest of the state, the percentage of pediatric dental claims paid by MCOs rose to over 96% (Figure 1). Missouri Medicaid beneficiaries under age 21 were mandated to enroll in managed care except those eligible for SSI, disabled children, children with special health needs or those in foster care (Missouri Health Net, 2020). As of 2017, Home State Health, Missouri Care, and United Healthcare administer the comprehensive MCO in Missouri (Medicaid.gov, 2017b). In this paper, counties in Missouri that transitioned to dental managed care prior to May 2017 were excluded from all analyses except Figure 1.

Effective October 1, 2017, Nebraska transitioned its pediatric Medicaid beneficiaries from FFS to managed care for dental services, contracting with Managed Care of North America (MCNA) to administer pediatric dental services (Medicaid.gov, 2017c). The transition established a dental home program to better coordinate relationships between providers and beneficiaries (Nebraska Department of Health and Human Services, n.d.). Prior to October 2017, less than 1% of total pediatric Medicaid dental claims were paid by managed care. After the transition occurred, over 99% of total dental claims in Nebraska were paid by a MCO (Figure 1). In Nebraska, MCNA is required to follow proper quality and accreditation guidelines (Medicaid.gov, 2017c).

When transitioning to managed care, Indiana and Missouri utilized a comprehensive contract (Medicaid.gov, 2017a, b), while Nebraska used a pre‐paid ambulatory health plan (PAHP) contract for dental services (Medicaid.gov, 2017c). Both comprehensive and PAHP contracts often contain network and quality requirements, require MCOs to facilitate outreach between providers and beneficiaries, and typically pay a risk‐based capitated payment regardless of whether or not beneficiaries receive services (MACPAC, n.d. (b)). MCO plans in both type of contracts would be at risk for losses if they make payments to providers in excess of what the state pays the MCO. On the other hand, if payments to providers are less than what the state pays the MCO, managed care plans would then be allowed to retain the profits as long as the MCO meets medical loss ratio requirements and reinvests any excess funds towards quality improvement (MACPAC, n.d. (b)). Therefore, MCOs in comprehensive and PAHP contracts may have an incentive to reduce utilization of dental services relative to FFS depending on the risk, payment, and provider arrangements used by the state with MCOs. These factors may also effect the relative “size” of the incentive when comparing similar types of contracts across states.

When transitioning from FFS to managed care, comprehensive and PAHP contracts could differentially impact utilization. Comprehensive contracts cover a broad range of services (e.g., medical services), in contrast to PAHP contracts which are usually carve‐outs for specialty services (e.g., dental services). While both comprehensive and PAHP contracts put financial risk on the MCOs, comprehensive contracts require the MCO to optimize a single capitated payment and contractual requirements across dental, medical, and other services while dental PAHP contracts have capitated payments and other contractual requirements that are solely tied to providing dental services. Because dental services are lower cost and more predictable than medical services, the more focused PAHP contracts may have less incentive to limit dental care use among Medicaid beneficiaries relative to comprehensive contracts.^4^

## 3 DATA

Currently, each state plus the District of Columbia submits monthly Medicaid claims and enrollment data to the Centers for Medicare and Medicaid Services (CMS) through the Transformed Medicaid Statistical Information System (T‐MSIS). Claims data include medical (inpatient and outpatient), pharmacy, long‐term care and dental claims. Dental claims are housed in the “other services” claims tables. The enrollment tables include demographic and location (state, county, zip code) characteristics for each beneficiary enrolled in Medicaid or CHIP. The claims and enrollment data available in T‐MSIS cover the universe of Medicaid and CHIP beneficiaries in each state, the District of Columbia and associated territories.

In T‐MSIS, the claims tables are split into separate header and line tables. The header tables include identifying information for whether the claim is paid on a FFS basis or by managed care. We used this identifying information to classify dental claims as FFS or managed care. The header and line tables are linkable through a unique claim identifier. From the other services line tables, we extracted all dental procedure codes that begin with the letter “D” (D0100‐D9999). These codes represent Codes on Dental Procedures and Nomenclature (CDT) (American Dental Association, 2020). While we have comprehensive information about the claims, a key limitation of the T‐MSIS claims data is that all payment information is masked for MCOs as per CMS requirements. Therefore, we examine utilization but not the prices paid or aggregate expenditures for those services.

At the county level in calendar years 2016 through 2018, we calculated two measures of utilization on a quarterly basis: the share of beneficiaries ages 0–18^5^ with a dental claim and the number of visits per 10,000 beneficiaries. These are the two main outcome variables we examined in our analysis. In supplementary analysis, we also examined select preventative and restorative services. We examined these procedure category outcome measures as one might expect that the effect of managed care on restorative dental care utilization to be smaller than the effect on other dental service categories. If a child has tooth pain or a cavity, a restorative procedure becomes medically necessary whereas fluoridation, diagnostic and preventive care could be more limited to conform to clinical dental guidelines. Additionally, restorative services typically cost more than preventive services (Meyerhoefer et al., 2019). The outcome variables examined in the supplementary analysis included: the share of enrolled beneficiaries with a diagnostic claim (D0100‐D0999), share of beneficiaries with a preventive claim (D1000‐D1999), share of beneficiaries with a prophylaxis claim (D1110, D1120), share of beneficiaries with a fluoridation claim (D1206, D1208), and the share beneficiaries with a restorative claim (D2000‐D2999). Prophylaxis (e.g., dental cleaning) and fluoridation procedures are considered two types of preventive services.^6^

Access to T‐MSIS Medicaid and CHIP dental claims data is part of a data use agreement approved by CMS (DUA RSCH‐2020‐5563: “The State of Oral Healthcare Use, Quality and Spending: Findings from Medicaid and CHIP Programs”).

## 4 EMPIRICAL STRATEGY

From 2016 to 2018, the three treatment states transitioned pediatric dental benefits in their Medicaid program from publicly administered FFS plans to privately administered managed care plans. We compared these states to 18 control states^7^ where Medicaid programs remained FFS (i.e., the percentage of total pediatric dental claims remained at or near 100% FFS) throughout the study period. This assures we did not include in the control group states that may have gradually shifted towards FFS or managed care over time. We also excluded from the regression analyses Missouri counties that transitioned to managed care prior to 2017. Our final analytic sample includes a balanced panel of 1086 counties where each county is observed for 12 quarters (*T* = 12) spanning from 2016 through 2018. Across all time periods, all counties from the three treatment states and 18 control states were pooled together in the main regression analyses.

Given the staggered introduction of dental managed care in the three treatment states, estimating a difference‐in‐differences specification via two‐way fixed‐effects (TWFE) could lead to biased policy estimates.^8^ As described by Goodman‐Bacon (2021), in a staggered policy intervention setting, the TWFE policy estimate is a weighted average of multiple comparisons: an early‐treated group versus a never‐treated group, a later‐treated group versus a never‐treated group, an early treated group versus a later‐treated group before it is treated, and a later treated group versus an earlier treated group after the early group is treated. It is this last comparison that can cause bias in the TWFE estimator when treatment effects vary over time or treatment cohort. For example, in our case, Indiana was first exposed to the treatment while Nebraska was the last to be exposed to treatment. One portion of the TWFE estimate consists of a 2 × 2 difference‐in‐differences comparison of Nebraska treated observations versus Indiana observations that have already been treated. Since Indiana was already on its treatment path, this particular 2 × 2 comparison could contaminate the composite TWFE estimate.

**FIGURE A1:**
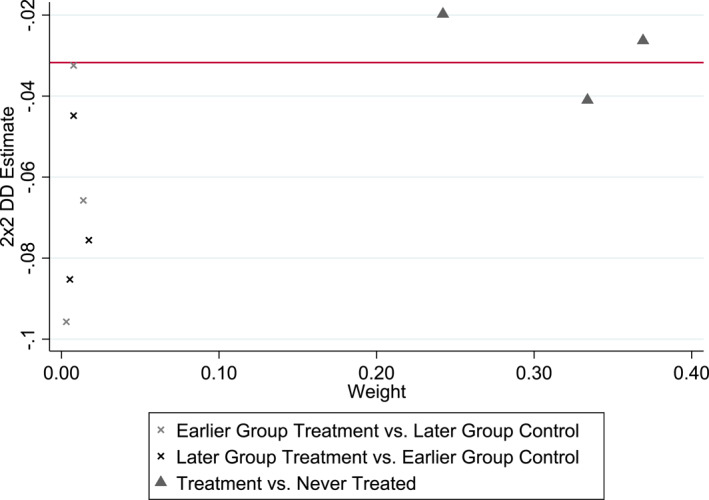
Bacon decomposition graph (difference‐in‐differences policy estimate of dental managed care on share of beneficiaries with a dental claim). *Source*: Goodman‐Bacon et al. (2019). Bacondecomp: Stata module for Decomposing difference‐in‐differences estimation with variation in treatment timing. https://ideas.repec.org/c/boc/bocode/s458676.html

To assess the amount of potential bias in the composite TWFE estimate, we estimated a detailed Goodman‐Bacon (2021) decomposition, the results of which are shown in Figure A1 and Table A2 for the share of beneficiaries with a dental claim. About 3% of the composite TWFE estimate comes from the potentially biased later treated versus earlier treated comparison. Although the timing groups (i.e., earlier treated vs. later treated and later treated vs. earlier treated) have a small overall weight, their average difference‐in‐differences estimate is about two times as large as the difference‐in‐differences estimate where a treated group is compared to the never treated group. This suggests potential heterogeneity in treatment effects across treatment cohorts and time.

To mitigate the potential bias in TWFE, Callaway and Sant’Anna (2021) proposed a difference‐in‐differences estimator that accounts for staggered treatment entry and many time periods, as in the case we examine in this paper. Callaway and Sant’Anna (2021) use outcome regression, inverse probability weighting and doubly robust methods to develop their difference‐in‐differences estimate. However, as shown by Wooldridge (2021), there is nothing inherently wrong with TWFE estimation in the presence of staggered entry except that it has been used in an overly restrictive manner when generating difference‐in‐differences policy estimates, which exposes estimates to bias as described by Goodman‐Bacon (2021). To mitigate the bias from comparing later treated groups to earlier treated groups, Wooldridge (2021) proposed a very flexible extended TWFE (ETWFE) regression estimator using interaction terms to account for staggered entry and multiple time periods in a panel data setting. The ETWFE regression estimator estimates separate average treatment effects on the treated (ATT) by treatment cohort and by time. By computing separate ATTs by treatment cohort and time, the ETWFE estimator does not compare later treated units to earlier treated units. The ATTs can then be aggregated to produce cohort specific and overall aggregate treatment effect estimates. This is a very similar approach to that used by Callaway and Sant’Anna (2021) except that standard regression methods are used to get the ATTs used for aggregation. We followed the methodology proposed by Wooldridge (2021) in what follows, though our results are robust to using the method proposed by Callaway and Sant’Anna (2021) and are discussed further in the Robustness Checks section.

In our set up, for county *c,* and periods *t* = 1,…,12, where *t* = 1 corresponds to 2016 quarter 1, we defined a vector of cohort indicators, ***d = *** (*d*_c5_, *d*_c6_, *d*_c8_) identified by when each county is exposed to treatment (e.g., dental managed care). Once counties get exposed to treatment, they remain treated. Never treated cohorts have treatment occur in period ∞. These cohort indicators do not vary over time within county. Instead they identify counties in cohorts (i.e., states), that eventually get exposed to treatment. In our application, the cohort indicators vary by state. The cohorts in our case are Indiana, Missouri and Nebraska. The cohort indicators for Indiana, the first treatment state, are set to period 5 (Quarter 1, 2017), period 6 (Quarter 2, 2017) for Missouri, and period 8 (Quarter 4, 2017) for Nebraska.^9^

In the presence of and conditional on a vector of time‐constant pre‐treatment covariates, ***X***_***c***_, we used a potential outcomes framework to define an average treatment effect on the treated (ATT), $\tau_{rt}$, for each treated cohort *r* = 5, 6, 8 and time periods *t* = 1,…,12

(1) $$\tau_{\text{rt}}\equiv E(y_{t}(r)-y_{t}(\infty )|d_{\text{cr}}=1,\;X_{c}]$$

where $y_{t}\left(r\right),\;r\in \left\{5,6,8\right\}$ is the potential outcome in time period *t* if an observation enters treatment in time period *r* and $y_{t}\left(\infty \right)$ is the potential outcome in time period *t* for an observation that is never treated. Included in the covariate vector $X_{c}$ are the following 2016 (pre‐reform) county‐level covariates: average county unemployment rate, dentists per capita, and median household income. These covariates vary by county.^10^ To identify cohort and time specific ATTs in the presence of a staggered policy intervention, we needed to assume linearity, a conditional no‐anticipation (CNA) assumption, and a conditional common/parallel trends (CCT) assumption.

$$(\text{CNA})\;E\left(y_{t}\left(r\right)-y_{t}\left(\infty \right)|d,\;X_{c}\right)=0\;\text{for}\;t<r$$

$$(\text{CCT})\;E\left(y_{t}\left(\infty \right)-y_{1}\left(\infty \right)\left|d,\;X_{c})=E(y_{t}\left(\infty \right)-y_{1}\left(\infty \right)\right|X_{c}\right)\;\text{for}\;t=2,\text{…},12$$

The CNA assumption holds if the potential outcomes are the same, $y_{t}\left(r\right)=y_{t}\left(\infty \right)$ prior to exposure to treatment. The CCT assumption says that conditional on covariates $X_{c}$ (unemployment rate, dentists per capita, median household income) the average trend in the control cohort, in every period relative to the first period, does not depend on treatment status, which is captured by ***d***. The CCT assumption implies that the average outcomes for treated groups and control groups (in this case never treated groups) would follow parallel paths in the absence of treatment.^11^ Assuming that all conditional expectations are linear in **X**_**c**_ **,** meaning that conditional on $d_{cr}=1$, $\dot{X}$_**c**_ **= X**_**c**_ **−**E(X_c_|$d_{cr}=1$), and that the CNA and CCT conditions hold, we can identify ATTs and estimate the following equation by pooled OLS to generate the ETWFE estimator

(2) $$E\left(y_{ct}|d,X_{c}\right)=\gamma +X_{c}\kappa +\sum_{r=5,6,8}\lambda_{r}d_{cr}+\sum_{r=5,6,8}\left(d_{cr}\cdot X_{c}\right)\zeta_{r}+\sum_{s=2}^{12}\theta_{s}fs_{t}+\sum_{s=2}^{12}\left(fs_{t}\cdot X_{c}\right)\pi_{t}+\sum_{r=5,6,8}\sum_{s=r}^{12}\tau_{rs}\left(d_{cr}\cdot fs_{t}\right)+\sum_{r=5,6,8}\sum_{s=r}^{12}\left(d_{cr}\cdot fs_{t}\cdot {\dot{X}}_{cr}\right)\rho_{rs}$$

where

(3) $${\dot{X}}_{\text{cr}}=X_{c}-N_{r}^{-1}\sum_{h=1}^{N}d_{\text{hr}}X_{h}$$

$fs_{t}$ are time fixed‐effects and *N*_*r*_ is the number of observations in cohort *r*. By centering the covariates around their cohort‐specific means in the last term in Equation (2) and described in Equation (3), we can recover the estimated $\tau_{rs}$ as the ATTs. The last estimated coefficient vector, $\rho_{rs},$ allows for heterogeneous treatment effects which are also called “moderating effects” by Wooldridge (2021). These coefficients can capture how treatment effects vary by various sub‐populations. In our context, treatment effects could vary by the economic health of a particular county which could be captured by the unemployment rate and median household income. The effect of the managed care reforms could also vary in counties that have a high or low supply of dentists as captured by county‐level dentists per capita.

The ATTs, estimated from Equation (2) can then be aggregated by cohort (e.g., state) and time to generate a cohort specific aggregate treatment effect

(4) $$\hat{\bar{\tau_{r}}}=\frac{1}{\left(12-r+1\right)}\sum_{t=r}^{12}\hat{\tau_{\text{rt}}}$$

and an overall aggregate treatment effect.

(5) $$\hat{\bar{\tau }}=\frac{1}{\left\{Overall\;Number\;of\;Estimated\;ATTs\right\}}\sum_{r=5,6,8}\sum_{t=r}^{12}\hat{\tau_{\text{rt}}}$$

The standard errors for the aggregated treatment effects were estimated using the delta method. Given that the data is aggregated to the county‐quarter level, we weighted the counties included in Equation (2) by their average Medicaid enrollment for children ages 0–18 from 2016 through 2018.

To test for violation of the common trends assumption, we estimated a version of Equation (2) by including cohort‐specific linear time trends.

(6) $$E\left(y_{ct}|d,X_{c}\right)=\gamma +X_{c}\kappa +\sum_{r=5,6,8}\lambda_{r}d_{cr}+\sum_{r=5,6,8}\left(d_{cr}\cdot X_{c}\right)\zeta_{r}+\sum_{s=2}^{12}\theta_{s}fs_{t}+\sum_{s=2}^{12}\left(fs_{t}\cdot X_{c}\right)\pi_{t}+\sum_{r=5,6,8}\sum_{s=r}^{12}\tau_{rs}\left(d_{cr}\cdot fs_{t}\right)+\sum_{r=5,6,8}\sum_{s=r}^{12}\left(d_{cr}\cdot fs_{t}\cdot {\dot{X}}_{cr}\right)\rho_{rs}+\sum_{r=5,6,8}\omega_{r}d_{cr}t$$

We then conducted the following joint test

(7) $$H_{0}:\omega_{5}=\omega_{6}=\omega_{8}=0\;$$

Rejection of the null hypothesis in Equation (7) indicates violation of the common trends assumption. Fortunately, if the common trends assumption is violated, including cohort‐specific time trends in Equation (6) would act as a correction of the violation of the common trends assumption.^12^

Because Indiana, Missouri and Nebraska implemented their transitions to dental MCOs statewide, we clustered standard errors by state. We only considered the Missouri counties that transitioned to dental managed care during the sample period, as the counties that transitioned to dental managed care prior to 2017 were not included in the sample. To mitigate potential issues in calculating clustered standard errors in a difference‐in‐differences setting with a small number of treated groups (Cameron et al., 2008; Conley & Taber, 2011), we implemented an ordinary wild bootstrap where each county‐quarter observation is used as its own “cluster” to generate *p*‐values and 95% confidence intervals as suggested by MacKinnon and Webb (2018) and Roodman et al. (2019).^13^

## 5 RESULTS

### 5.1 Summary statistics

In Table 2, we present summary statistics for the three states that transition to a dental MCO prior to their transition and our set of control states prior to the first observed MCO transition in our sample. For overall utilization (share of beneficiaries with a dental claim and dental visits per 10,000 beneficiaries) and across the various service categories (diagnostic, preventive, restorative, prophylaxis and fluoridation), pre‐reform utilization in Missouri was typically lower than in the other treatment states and the control states. Conversely, Nebraska prior to its transition to dental managed care had higher dental utilization levels than the other treatment and control states. The order of this pattern holds when one examines the various dental procedure categories (diagnostic, preventive, restorative, prophylaxis and fluoridation).

**TABLE 2:** Summary statistics

	Indiana	Missouri	Nebraska	Control states
	Pre‐reform			Prior to January 1, 2017 (Indiana reform)
Dental care use dependent variables				
Share of beneficiaries with a dental claim	0.225	0.185	0.273	0.250
	(0.020)	(0.026)	(0.032)	(0.067)
Dental visits per 10,000 beneficiaries	2885.54	2436.01	3529.5	3333.321
	(250.687)	(396.393)	(424.3)	(994.299)
Share of beneficiaries with a diagnostic claim	0.204	0.154	0.233	0.212
	(0.021)	(0.022)	(0.031)	(0.057)
Share of beneficiaries with a preventive claim	0.193	0.145	0.236	0.204
	(0.021)	(0.023)	(0.034)	(0.055)
Share of beneficiaries with a prophylaxis	0.185	0.131	0.206	0.181
	(0.021)	(0.022)	(0.033)	(0.048)
Share of beneficiaries with a fluoridation	0.177	0.130	0.213	0.189
	(0.022)	(0.024)	(0.035)	(0.052)
Share of beneficiaries with a restorative claim	0.055	0.051	0.059	0.054
	(0.007)	(0.012)	(0.010)	(0.019)
Individual beneficiary characteristics				
Age	8.659	8.857	8.250	8.671
	(5.382)	(5.291)	(5.243)	(5.367)
Female	0.487	0.486	0.489	0.489
	(0.500)	(0.500)	(0.500)	(0.500)
2016 pre‐reform county‐level covariates				
2016 county unemployment rate	4.574	5.159	3.147	5.010
	(0.800)	(1.425)	(0.389)	(1.376)
2016 county median household income in $000s	51.447	41.334	56.962	58.699
	(8.989)	(6.073)	(6.654)	(17.633)
2016 county dentists per capita	48.628	37.450	67.660	57.162
	(15.781)	(21.031)	(21.144)	(26.214)

*Note*: Standard deviation in parentheses. Summary statistics (means and standard deviations) calculated over the period prior to the transition to dental managed care in each state. Summary statistics for dental care utilization and county characteristics calculated based on data aggregated to the county and quarter level and weighted based on average county enrollment in Medicaid among children ages 0–18 from 2016 through 2018. Summary statistics for beneficiary characteristics based on beneficiary level data. Statistics include data from three treatment states (Indiana, Missouri and Nebraska) and 18 control states which were fully fee‐for‐service during the study period. Dates states transitioned to managed care are: Jan. 1, 2017 (Indiana); May 1, 2017 (Missouri); Oct. 1, 2017 (Nebraska).

*Source*: 2016–2018 T‐MSIS Medicaid and CHIP Analytic Claims Data from children ages 0–18. Unemployment rate: U.S. Bureau of Labor Statistics. Dentists per capita: American Dental Association. Median Household Income: United States Census.

The age and gender distribution was also very similar across the treatment and control states. In 2016, median household income was lower in the treatment states than in the control states. The number of dentists per capita was also lowest in Missouri (37.5) and highest in Nebraska (67.7). In the control states, the average county‐level number of dentists per capita was 57.2.

### 5.2 Main results

**FIGURE A2:**
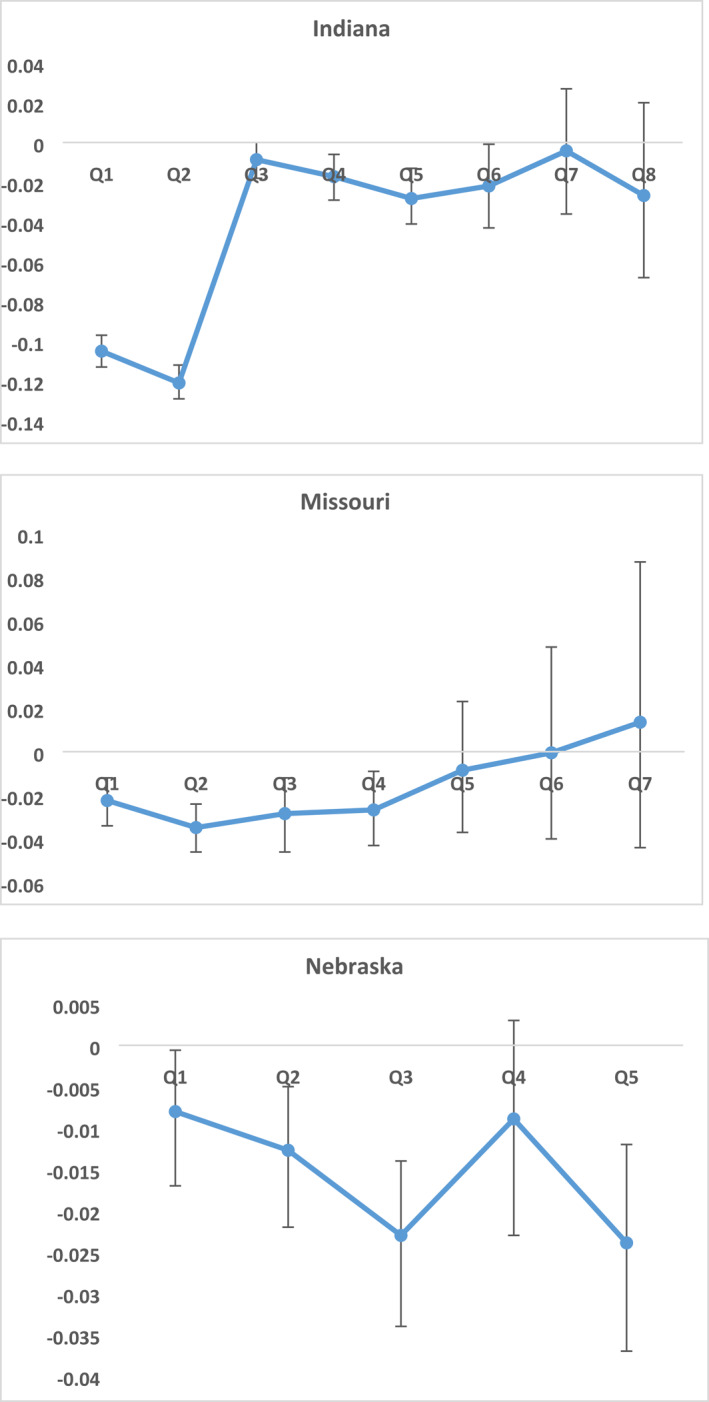
Plot of state and post‐period specific ATTs. Share of beneficiaries with a dental claim. Quarter‐specific ATTs estimated by ETWFE using Equation (2). Pre‐reform 2016 covariates in regression include county‐level unemployment rate, dentists per capita and real median household income in 2016 dollars. ATT, Average Treatment Effect on the Treated; ETWFE, Extended two‐way fixed effects. 95% confidence intervals generated from wild bootstrap with 9999 replications. Twenty‐one states in each regression include the three treatment states (Indiana, Missouri and Nebraska) and 18 states which were fully fee‐for‐service during the study period. ***p < 0.01; **p < 0.05; *p < 0.1. Dates states transitioned to managed care are: Jan. 1, 2017 (Indiana); May 1, 2017 (Missouri); Oct. 1, 2017 (Nebraska). Number of observations: 13,032. Number of counties: 1086. *Source*: 2016–2018 T‐MSIS Medicaid and CHIP Analytic Claims Data from children ages 0–18. Unemployment rate: U.S. Bureau of Labor Statistics. Dentists per capita: American Dental Association. Median Household Income: United States Census

**FIGURE A3:**
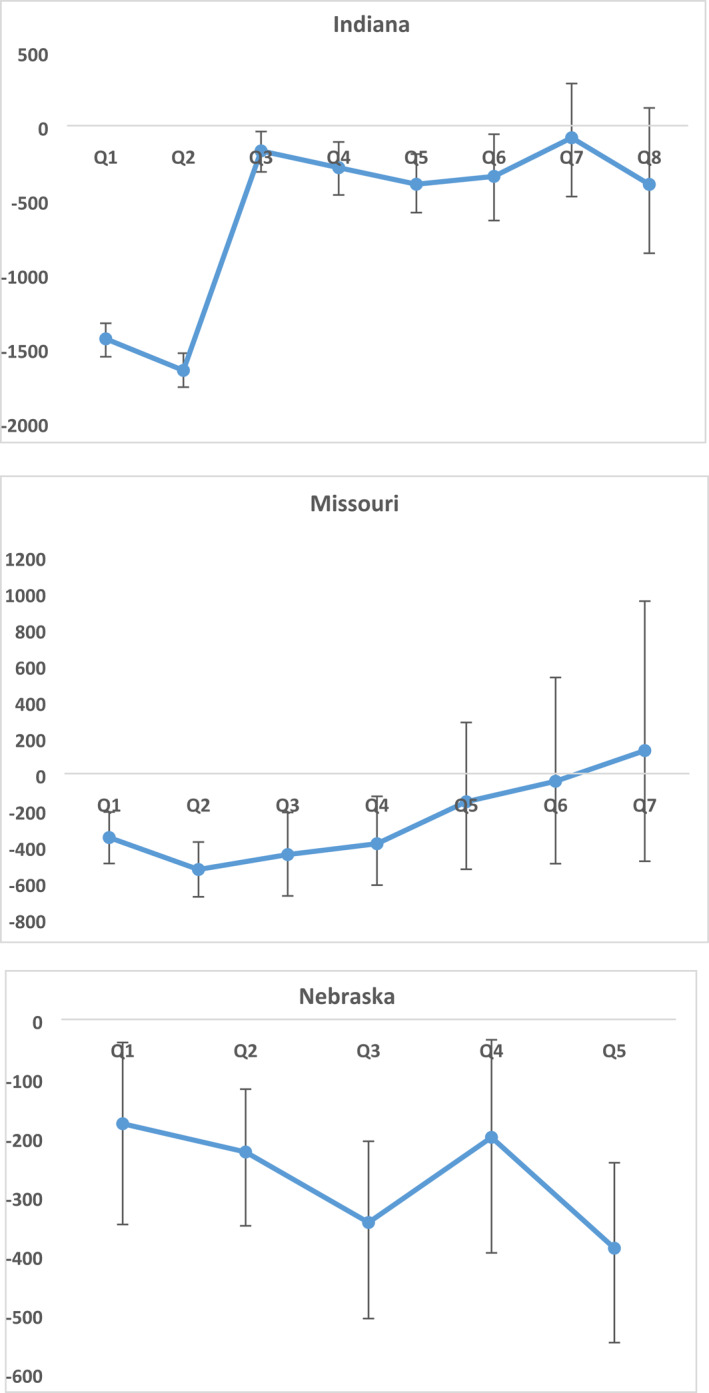
Plot of state and post‐period specific ATTs. Visits per 10,000 beneficiaries. Quarter‐specific ATTs estimated by ETWFE using Equation (2). Pre‐reform 2016 covariates in regression include county‐level unemployment rate, dentists per capita and real median household income in 2016 dollars. ATT, Average Treatment Effect on the Treated; ETWFE, Extended two‐way fixed effects. 95% confidence intervals generated from wild bootstrap with 9999 replications. Twenty‐one states in each regression include the three treatment states (Indiana, Missouri and Nebraska) and 18 states which were fully fee‐for‐service during the study period. ***p < 0.01; **p < 0.05; *p < 0.1. Dates states transitioned to managed care are: Jan. 1, 2017 (Indiana); May 1, 2017 (Missouri); Oct. 1, 2017 (Nebraska). Number of observations: 13,032. Number of counties: 1086. *Source*: 2016–2018 T‐MSIS Medicaid and CHIP Analytic Claims Data from children ages 0–18. Unemployment rate: U.S. Bureau of Labor Statistics. Dentists per capita: American Dental Association. Median Household Income: United States Census.

Table 3 reports the cohort and time specific ATTs for Indiana, Missouri and Nebraska in addition to the cohort specific treatment effects. Corresponding coefficient plots with 95% confidence intervals are shown in Figures A2 and A3 for the share of beneficiaries with a dental claim and the number of dental visits per 10,000 beneficiaries outcomes, respectively. In the first two quarters after its transition to dental managed care, Indiana had a large decline in dental care utilization. The share of beneficiaries with a dental claim declined by 10.5%–12% points (*p* < 0.01) or by about 47%–54% in the first 6  months of the managed care transition in Indiana. Utilization rebounded after the 6 ‐month mark, but remained below pre‐reform levels. Specifically, in the fifth and sixth quarters following the dental managed care implementation in Indiana, the share of beneficiaries with a dental claim fell 2.2%–2.8% points (*p* < 0.05) or by 10%–13.5% relative to the pre‐reform period. This general pattern is also present in the number of dental visits per 10,000 beneficiaries. Overall, in Indiana, the share of beneficiaries with a dental claim declined by 4.2% points (*p* < 0.01) or by about 18%, and the number of visits per 10,000 beneficiaries declined by 594 visits (*p* < 0.01) or by about 20.6% relative to the pre‐reform period.

**TABLE 3:** ATTs. Policy effects of transition from FFS to dental managed care on dental care utilization by quarter

	*Indiana*		*Missouri*		*Nebraska*	
Dependent variable	Share of beneficiaries with a dental claim	Visits per 10,000 beneficiaries	Share of beneficiaries with a dental claim	Visits per 10,000 beneficiaries	Share of beneficiaries with a dental claim	Visits per 10,000 beneficiaries
Aggregated ATT	−0.0415***	−594.3***	−0.0154	−255.3*	−0.0153***	−267.0***
	(0.005)	(65.70)	(0.006)	(88.65)	(0.003)	(43.73)
	[−0.054, −0.027]	[−769.7, −399.4]	[−0.036, 0.005]	[−532.7, 19.21]	[−0.024, −0.007]	[−385.2, −151.9]
Quarter 1 ATT	−0.105***	−1437***	−0.0224***	−353.0***	−0.00802**	−177.0**
	(0.00269)	(40.85)	(0.00330)	(44.75)	(0.00313)	(54.85)
	[−0.113, −0.097]	[−1558, −1322]	[−0.034, −0.012]	[−496, −212.1]	[−0.017, −0.00006]	[−347.9, −39.12]
Quarter 2 ATT	−0.121***	−1650***	−0.0348***	−529.4***	−0.0127***	−225.0***
	(0.00310)	(42.46)	(0.00322)	(49.20)	(0.00288)	(40.91)
	[−0.129, −0.112]	[−1763, −1534]	[−0.046, −0.024]	[−680.2, −377.9]	[−0.022, −0.005]	[−350.2, −118.3]
Quarter 3 ATT	−0.00864**	−170.0**	−0.0284***	−447.0***	−0.0230***	−344.6***
	(0.00315)	(49.55)	(0.00465)	(69.62)	(0.00378)	(56.23)
	[−0.018, −4.4e−06]	[−311.3, −39.47]	[−0.046, −0.012]	[−675.3, −214.3]	[−0.034, −0.014]	[−507.6, −206.8]
Quarter 4 ATT	−0.0172**	−283.1***	−0.0267**	−386.9**	−0.00891	−200.1**
	(0.00394)	(61.28)	(0.00547)	(79.13)	(0.00450)	(64.59)
	[−0.029, −0.006]	[−467, −110]	[−0.043, −0.009]	[−615.8, −124.5]	[−0.023, 0.003]	[−396.2, −34.69]
Quarter 5 ATT	−0.0282***	−394.8***	−0.00851	−155.3	−0.0239***	−388.2***
	(0.00455)	(66.52)	(0.00836)	(117.9)	(0.00457)	(58.72)
	[−0.041, −0.013]	[−586.1, −191.5]	[−0.037, 0.023]	[−528.2, 282.4]	[−0.037, −0.012]	[−548.3, −243.2]
Quarter 6 ATT	−0.0219**	−341.8**	−0.000511	−42.33	‐‐	‐‐
	(0.00645)	(92.22)	(0.0102)	(136.8)		
	[−0.043, −0.0009]	[−639.7, −57.64]	[−0.040, 0.048]	[−497.2, 530.6]		
Quarter 7 ATT	−0.00421	−81.32	0.0135	127.1	‐‐	‐‐
	(0.00766)	(104.3)	(0.0126)	(166.8)		
	[−0.036, 0.027]	[−477.6, 284.1]	[−0.044, 0.087]	[−484, 952.4]		
Quarter 8 ATT	−0.0267	−396.0*	‐‐	‐‐	‐‐	‐‐
	(0.00907)	(121.2)				
	[−0.068, 0.020]	[−859.9, 119.7]				

*Note*: Number of observations: 13,032. Number of counties: 1086. Robust clustered standard errors by state in parentheses. 95% confidence intervals and *p*‐values generated from wild bootstrap with 9999 replications in brackets. Pre‐reform 2016 covariates include county‐level unemployment rate, dentists per capita and real median household income in 2016 dollars. Twenty one states in each regression include the three treatment states (Indiana, Missouri and Nebraska) and 18 states which were fully fee‐for‐service during the study period.

Abbreviation: ATT, Average Treatment Effect on the Treated.

***p < 0.01; **p < 0.05; *p < 0.1. Dates states fully transitioned to managed care: Indiana—January 01, 2017, Missouri—January 05, 2017, Nebraska—January 10, 2017.

*Source*: 2016–2018 T‐MSIS Medicaid and CHIP Analytic Claims Data from children ages 0–18. Unemployment rate: Bureau of Labor Statistics. Dentists per capita: American Dental Association. Median Household Income: United States Census.

In the Missouri counties that transitioned to dental managed care in May 2017, the share of beneficiaries with a dental claim fell by 2.2%–3.5% points (12.1%–18.8%) in the first four quarters following the dental managed care transition relative to the pre‐reform level. In the following three quarters, relative to the pre‐reform baseline, there was no statistically significant change in dental care utilization. There was a statistically significant decline of 353–529 visits per 10,000 beneficiaries in the first four quarters in the first four quarters in Missouri following its transition to dental manage care, but visits rebounded 1  year after the transition. Across the Missouri counties that transitioned to dental managed care, the share of beneficiaries with a dental claim declined by 1.5% points, a statistically insignificant decline. The number of dental visits per 10,000 beneficiaries declined by 255 visits (*p* < 0.10) or by 10.5% relative to the pre‐reform period.

In Nebraska, utilization fell more modestly than in Indiana and Missouri, but the declines were statistically significant. In the first five quarters following its transition to dental managed care, relative to its baseline pre‐reform average, the share of beneficiaries with a dental claim fell by 0.8%–2.4% points (3.1%–8.8%). Visits per 10,000 beneficiaries fell by 177–388 visits (5%–11%) in the first five quarters following the reform. Overall, in Nebraska, the share of beneficiaries with a dental claim declined by 1.5% points (*p* < 0.01) or by 5.9% relative to the state's pre‐reform period and the number of visits per 10,000 beneficiaries declined by 267 visits (*p* < 0.01) or by 7.6%.

Table 4 reports the aggregated overall treatment effect (5) as derived from Equation (2). Pooling the three states together, the share of beneficiaries with a dental claim declined by 2.6% points (*p* < 0.01) and visits per 10,000 beneficiaries fell by 394 (*p* < 0.01).

**TABLE 4:** Aggregated ATTs. Overall association between transition from fee‐for‐service to managed care and dental care utilization

	Share of beneficiaries with a dental claim	Visits per 10,000 beneficiaries
Aggregated ATT across 3 treatment states	−0.0258***	−393.8***
	(0.005)	(65.68)
	[−0.038, −0.014]	[−558.4, −230.1]

*Note*: Aggregated ATT across 3 treatment states estimated using Equation (5). Standard errors in parentheses. The standard errors were estimated using the delta method. 95% confidence intervals in brackets. *p* values and 95% confidence intervals generated from wild bootstrap with 9999 replications. Number of observations: 13,032. Number of counties: 1086.

Abbreviation: ATT, Average Treatment Effect on the Treated.

***p < 0.01; **p < 0.05; *p < 0.1.

*Source*: 2016–2018 T‐MSIS Medicaid and CHIP Analytic Claims Data from children ages 0–18. Unemployment rate: U.S. Bureau of Labor Statistics. Dentists per capita: American Dental Association. Median Household Income: United States Census.

To assure our results were not driven by a violation in the common trends assumption, we re‐estimated the model with cohort specific linear time trends (Table 5). For all dependent variables, the joint tests do not reject the null hypothesis of common trends. Given that the joint test does not reject the null hypothesis for any dependent variable, we did not include a cohort‐specific linear time trend in any final specification. There is weak evidence of a violation of the common trends assumption in Missouri for visits per 10,000 beneficiaries, but the coefficient on the linear time trend in Missouri is only marginally significant (*p* < 0.10).

**TABLE 5:** Joint test for violation of common trends assumption

	Share of beneficiaries with a dental claim	Visits per 10,000 beneficiaries	Share of beneficiaries with a dental diagnostic claim	Share of beneficiaries with a dental preventive claim	Share of beneficiaries with a prophylaxis claim	Share of beneficiaries with a fluoridation claim	Share of beneficiaries with a dental restorative claim
Indiana cohort × linear trend	−0.000507	−9.418	−0.000647	−0.000181	2.44e−05	−0.000102	2.58e−05
	(0.000952)	(12.84)	(0.000851)	(0.000876)	(0.000569)	(0.000849)	(0.000201)
	[−0.003, 0.002]	[−44.91, 25.37]	[−0.003, 0.002]	[−0.003, 0.002]	[−0.002, 0.002]	[−0.003, 0.003]	[−0.0007, 0.0007]
Missouri cohort × linear trend	−0.00125	−35.77*	−0.000887	−0.00152	−0.000427	−0.00149	−0.00107
	(0.000917)	(13.63)	(0.000835)	(0.000809)	(0.000649)	(0.000751)	(0.000351)
	[−0.004, 0.002]	[−78.23, 5.808]	[−0.004, 0.002]	[−0.004, 0.001]	[−0.003, 0.002]	[−0.004, 0.001]	[−0.003, 0.0003]
Nebraska cohort × linear trend	−0.00107	−18.03	−0.000574	−0.000923	−0.000430	−0.000747	−0.000484
	(0.000735)	(11.00)	(0.000600)	(0.000610)	(0.000559)	(0.000569)	(0.000428)
	[−0.003, 0.001]	[−48.68, 11.48]	[−0.003, 0.001]	[−0.003, 0.001]	[−0.003, 0.002]	[−0.003, 0.001]	[−0.002, 0.001]
Joint test for violation for common trends assumption	*F* = 0.84; *p*‐value = 0.82	*F* = 3.08; *p*‐value = 0.33	*F* = 0.42; *p*‐value = 0.94	*F* = 2.00; *p*‐value = 0.62	*F* = 0.27; *p*‐value = 0.98	*F* = 2.32; *p*‐value = 0.60	*F* = 10.89; *p*‐value = 0.13

*Note*: Cohort (e.g., state) specific coefficients on linear time trends estimated by ETWFE using Equation (6). Inclusion of cohort‐specific linear time trends used to test for violation of the common trends assumption. Pre‐reform 2016 covariates in regression include county‐level unemployment rate, dentists per capita and real median household income in 2016 dollars. Cluster robust standard errors by state in parentheses. *p*‐values and 95% confidence intervals generated from wild bootstrap with 9999 replications. Twenty‐one states in each regression include the three treatment states (Indiana, Missouri and Nebraska) and 18 states which were fully fee‐for‐service during the study period.

Abbreviation: ETWFE, extended two‐way fixed effects.

***p < 0.01; **p < 0.05; *p < 0.1. Dates states transitioned to managed care are: Jan. 1, 2017 (Indiana); May 1, 2017 (Missouri); Oct. 1, 2017 (Nebraska). Number of observations: 13,032. Number of counties: 1086.

*Source*: 2016–2018 T‐MSIS Medicaid and CHIP Analytic Claims Data from children ages 0–18. Unemployment rate: U.S. Bureau of Labor Statistics. Dentists per capita: American Dental Association. Median Household Income: United States Census.

### 5.3 Robustness checks

To examine the robustness of the cohort and quarter specific ATTs from the Wooldridge (2021) estimator, we also estimated a specification using the Callaway and Sant’Anna (2021) difference‐in‐differences estimator using doubly robust estimation as specified in Callaway and Sant’Anna (2021) and Sant’Anna and Zhao (2020). The cohort and quarter specific ATTs for Indiana, Missouri and Nebraska are presented in Table A3 and the aggregate ATT across all 3 treatment states and post‐periods are presented in Table A4.

Overall, our results when we apply the Callaway and Sant’Anna (2021) estimator are very similar to the main specification and imply qualitatively similar conclusions. The cohort and quarter specific ATTs for Indiana and Missouri are identical in sign and very close in magnitude to the main specification using the Wooldridge (2021) estimator. For Nebraska, the cohort and quarter specific ATTs are moderately larger in magnitude compared to the Wooldridge (2021) estimator. For the aggregated overall treatment effect, the traditional TWFE (Table A1) and Callaway and Sant’Anna estimator (Table A4) imply larger declines in the share of beneficiaries with a dental claim and visits per 10,000 beneficiaries than the Wooldridge estimator (Table 4), with all three approaches yielding statistically significant effects. In conclusion, our results appear to be robust when applying the Callaway and Sant’Anna (2021) estimator and the traditional TWFE estimator. This may be due to the fact that there are few treated cohorts in our estimation sample and few cases when later treated units are compared to early treated units as shown by the Goodman‐Bacon (2021) decomposition (Figure A1 and Table A2).

### 5.4 Dental procedure categories

To better understand how utilization declined in Indiana, Missouri and Nebraska following the transition to dental managed care, we also examined the change in utilization by dental procedure category. The aggregated ATTs over the entire post‐period imply the share of beneficiaries with a diagnostic claim (Table A5) significantly declined by 4% and 1% points in Indiana and Nebraska, respectively. In all three states, there were statistically significant declines in the share of beneficiaries with a diagnostic claim in the first few quarters.

Changes in preventive dental care utilization also followed a similar pattern in the three states that fully transitioned to dental managed care in 2017 (Table A6). Overall, the share with a preventive dental care declined by 3.8% points (19.7%) in Indiana (*p* < 0.01), 1.7% points (11.9%) in Missouri (*p* < 0.10), and 1.1% points (5.0%) in Nebraska (*p* < 0.01). These results are confirmed by the changes in the share of beneficiaries that receive prophylaxis and fluoridation dental care services (Tables A7 and A8). Interestingly, while the effects become smaller over time, there were still quarters one full year after the transition in which utilization was below pre‐reform levels in all states.

Finally, the share of members receiving restorative dental care services experienced smaller declines than overall, diagnostic, and preventative dental services (Table A9). In fact, only the aggregated ATT with respect to dental restorative utilization for Indiana was marginally statistically significant at the 10% level. Most of the decline in restorative dental care services in Indiana occurred in the first 6 months following the state's transition to dental managed care. In the subsequent quarters, restorative dental care utilization in Indiana returned to near pre‐reform levels. Given that restorative dental care services are medically necessary when a child has tooth pain or cavities, it should not be surprising that the dental managed care transition had less of an impact on these services than on diagnostic or preventive dental care services, which may be easier for a managed care entity to limit the use of or ration.

## 6 CONCLUSION

Between 2016 and the end of 2018, Indiana, Missouri and Nebraska transitioned their Medicaid pediatric dental benefits from a FFS model to a private managed care system. This study examines dental service utilization patterns associated with this transition. Utilizing an extended TWFE approach as proposed by Wooldridge (2021), we estimated ATTs for each state over time and calculated aggregate treatment effects across the three treatment states. The main analysis examined the proportion of pediatric Medicaid beneficiaries that had a dental claim and the number of dental visits per 10,000 beneficiaries.

The theoretically predicted effect of the transition to managed care is mixed. Managed care organizations could enact strategies to decrease utilization in order to assure dental expenditures by the MCO are below the payments received from the state. Conversely, the MCO may also implement strategies that increase utilization, especially if MCOs promote the use of preventative services that could reduce later need for more costly restorative services. Our empirical results find that relative to states that continued to provide pediatric dental services exclusively on a FFS basis, there is evidence of a decline in dental services utilization following the transition to managed care. Pediatric dental care use among publicly insured children in Indiana declined by about 18% in the 2 years after the state's transition to dental managed care and by about 12–19% in the counties that transitioned to managed care in Missouri in the first year after the state's transition from FFS. The decline in utilization was more modest in Nebraska, but still fell by about 6%.

The pattern in Indiana is particularly striking, with large declines in the first few months and then more modest declines in utilization 6 months after the transition to managed care.^14^ A qualitative interview with a local official in Indiana suggested that the transition was not smooth and the pattern seen in 2017 was due to complications in the implementation process. For example, in 2017, Indiana changed its provider credentialing systems and many dentists were not able to properly submit claims to the dental MCO during the first few months following the transition to managed care. While this may explain the decline in utilization in the first 6 months after the transition, the decline in pediatric Medicaid dental care utilization in Indiana persisted through much of 2018. It is possible that some dental providers stopped treating Medicaid beneficiaries due to difficulty in dealing with the administrative aspects of dental managed care, but this requires further research beyond the scope of this paper. It is difficult to tease out whether the persistent decrease in dental care utilization in Indiana is due to problems with claims processing or information technology systems or MCOs having an incentive to limit spending. It may be a combination of both.

All states saw declines in utilization of dental services, with the largest declines in Indiana. The magnitude of the declines in Missouri and Nebraska were similar (even though the effect for Missouri was not always statistically significant). While our empirical analysis cannot test the exact reason for this pattern, one hypothesis is that states with more generous Medicaid FFS reimbursement have better access to care and beneficiaries in these states might be more affected by a transition to managed care (Layton et al., 2019). Our results suggest that this may be true. In 2016, Medicaid FFS rates in Indiana were at 69.2% of commercial rates; in Missouri Medicaid FFS rates were at 50% of commercial rates; and in Nebraska FFS Medicaid rates were at 59% of commercial rates for pediatric dental services (Gupta et al., 2017). This result is consistent with outcomes being worse in states that had more generous FFS rates. However, given that most of the decline in dental care utilization in Indiana occurred in the first two quarters of the dental managed care transition, possibly due to problems with the implementation process in the state, it is difficult to make any strong connection between pre‐existing FFS provider rates and subsequent outcomes under managed care.

In conclusion, as states move to transition more Medicaid benefits, such as long‐term care, behavioral health, and dental services to managed care, additional research is warranted to assure beneficiaries can utilize services and care quality is not diminished. Our study shows that the recent transitions from FFS to managed care in the carved‐out service of dental care tend to result in lower utilization rates. Furthermore, the experience of Indiana shows that without properly implementing administrative and information technology systems, the transition to dental managed care can result in significant service delivery disruption for beneficiaries. While this reduction in utilization can be seen as a negative, our study is limited by not having measures of quality. If dental MCOs are better able to coordinate contact of beneficiaries with dental providers, this could result in better dental outcomes that require fewer dental visits, though this is unlikely. If this were the case, we would see fewer overall visits, fewer visits for restorative care, but no change in preventative visits. Instead, we found that dental care utilization declined across diagnostic and preventive service categories. Furthermore, we did not have payment information. This limited our ability to compare expenditures between public and private provision of pediatric Medicaid dental services. Our results highlight that MCOs may reduce utilization of dental services, but additional research is needed to understand the quality and cost implications of this decreased utilization.

## CONFLICT OF INTEREST

The authors declare no conflict of interest.

## Data Availability

The data that support the findings of this study are available from the Centers for Medicare and Medicaid Services through the Research Data Assistance Center. (https://www.medicaid.gov/medicaid/data-systems/macbis/medicaid-chip-research-files/transformed-medicaid-statistical-information-system-t-msis-analytic-files-taf/index.html) (https://urldefense.com/v3/__https://resdac.org/__;!!N11eV2iwtfs!te0zTKjGtBNV4QDzHDvzRFo_73DZsjj03ELaG7Ryift67I0w9TN2ZmWYuhYtuxwSN4fVjVWvuw$). Restrictions apply to the availability of these data, which were used under license for this study.

## ENDNOTES

^1^
To simplify the exposition throughout this paper, we refer only to Medicaid even though some children are enrolled via the Children's Health Insurance Program (CHIP).

^2^
According to our analysis of T‐MSIS Medicaid dental claims data, in 2016, Tennessee had zero percent of dental claims paid for by a MCO. Also, enrollment in pediatric dental MCO in Tennessee dropped to zero in January 2016. Hence, unlike the report from the American Dental Association, we classify Tennessee as having a fee‐for‐service pediatric Medicaid dental benefit.

^3^
Arkansas transitioned to dental managed care on January 1, 2018 for its pediatric Medicaid population (Medicaid.gov, 2018b). However, due to poor data quality in Arkansas in the T‐MSIS claims tables, we decided not to evaluate the state's transition to dental managed care. In 2016, Arkansas had substantially lower pediatric dental visits per enrolled member compared to other states. We do not attribute these differences to policy discrepancies between Arkansas and other states as there were no relevant policy changes that we are aware of in Arkansas prior to 2018. Instead, we attribute these differences in dental care use due to under‐reporting of dental claims in Arkansas in 2016. Including Arkansas in our specification would generate a significant differential pre‐trend in our analysis, making it more difficult to evaluate the policy reform in the state. Hence, out of an abundance of caution, we dropped Arkansas from the analysis.

^4^
None of the states we considered in our analysis transitioned from FFS to a primary care case management (PCCM) dental MCO. Under such a system, MCOs focus on development of care plans, quality improvement, and support for providers (MACPAC, n.d. (b)). Dental care utilization may increase if states transitioned to a PCCM dental MCO. However, under such a system, beneficiaries need to get referrals from primary care providers to see specialists, which could limit utilization of specialized services.

^5^
Although the mandatory EPDST pediatric dental benefit covers children ages 0–20, we restricted our analysis to beneficiaries ages 0–18 because the transition from FFS to managed care in Indiana only covered children up to age 19. Nonetheless, our results are robust to the inclusion of 19 and 20 year olds.

^6^
It is recommended that children receive fluoride varnish every 3–6 months to primary and permanent teeth (Weyant et al., 2013).

^7^
The control states are Alabama, Alaska, Connecticut, Delaware, Hawaii, Massachusetts, Maryland, Maine, Montana, North Carolina, North Dakota, New Hampshire, Oklahoma, South Carolina, South Dakota, Tennessee, Virginia and Washington State.

^8^
We also estimated the model using a standard difference‐in‐differences specification and came to qualitatively similar conclusions as those implied by our preferred estimator. See Table A1.

^9^
For counties in Indiana, *d*_c5_ = 1. For counties in Missouri, *d*_c6_ = 1. For counties in Nebraska, *d*_c8_ = 1.

^10^
In their setup, Callaway and Sant'Anna (2021) and Wooldridge (2021) condition on time‐constant pre‐reform covariates. Although county unemployment rate, dentist per capita, and median household income naturally vary over time, they are constructed and included in the regression such that they do not vary over time.

^11^
We test for violation of the common trends assumption conditional on $X_{c}$ and we generally find no evidence that the common trends assumption is violated.

^12^
If the null hypothesis of common trends is not rejected, including cohort‐specific linear time trends in Equation (2) would not be advisable as this would introduce unnecessary collinearity into the regression estimates.

^13^
Roodman et al. (2019) applied an ordinary wild bootstrap using the empirical example provided by Conley and Taber (2011).

^14^
In the data, Indiana did not see a drop in enrollees after the transition to dental managed care.

## Appendix

**TABLE A1:** Traditional two‐way fixed effects policy effect of transition from FFS to dental managed care on dental care utilization

	Share of beneficiaries with a dental claim	Visits per 10,000 beneficiaries
TWFE policy estimate	−0.032*	−472.77**
	(0.0087)	(113.86)
	[−0.074, 0.005]	[−1007, −1.79]

*Note* **:** Number of observations: 13,032. Number of counties: 1086. 95% confidence intervals generated from wild bootstrap with 9999 replications in brackets. Twenty‐one states in each regression include the three treatment states (Indiana, Missouri and Nebraska) and 18 states which were fully fee‐for‐service during the study period.

Abbreviations: FFS, fee‐for‐service; TWFE, two‐way fixed‐effects.

****p* < 0.01; ***p* < 0.05; **p* < 0.1. *p*‐values generated from wild bootstrap with 9999 replications. Dates states transitioned to managed care are: Jan. 1, 2017 (Indiana); May 1, 2017 (Missouri); Oct. 1, 2017 (Nebraska).

*Source*: 2016–2018 T‐MSIS Medicaid and CHIP Analytic Claims Data from children ages 0–18.

**TABLE A2:** Detailed Bacon‐decomposition. Timing groups. (Difference‐in‐differences policy estimate of dental managed care on share of beneficiaries with a dental claim)

Comparison	Weight	Average difference‐in‐differences
Earlier treated versus Later treated	0.025	−0.059
Later treated versus Earlier treated	0.03	−0.069
Treated versus Never treated	0.945	−0.03
Composite difference‐in‐differences		−0.032

*Source*: Goodman‐Bacon et al. (2019). Bacondecomp: Stata module for Decomposing difference‐in‐differences estimation with variation in treatment timing. https://ideas.repec.org/c/boc/bocode/s458676.html.

**TABLE A3:** Callaway and Sant’Anna (2021) ATTs. Policy effects of transition from FFS to dental managed care on dental care utilization by quarter

	Indiana		Missouri		Nebraska	
	Share of beneficiaries with a dental claim	Visits per 10,000 beneficiaries	Share of beneficiaries with a dental claim	Visits per 10,000 beneficiaries	Share of beneficiaries with a dental claim	Visits per 10,000 beneficiaries
Aggregated ATT	−0.0414***	−590.6***	−0.0135***	−207.6***	−0.0273***	−400.7***
	(0.00393)	(53.44)	(0.00363)	(50.95)	(0.00326)	(58.48)
	[−0.0491, −0.0337]	[−695.3, −485.8]	[−0.0206, −0.00640]	[−307.4, −107.7]	[−0.0337, −0.0209]	[−515.3, −286.1]
Quarter 1 ATT	−0.104***	−1423***	−0.0170***	−258.6***	−0.0220***	−331.8***
	(0.00291)	(40.52)	(0.00218)	(34.68)	(0.00360)	(58.69)
	[−0.109, −0.0980]	[−1503, −1344]	[−0.0213, −0.0128]	[−326.6, −190.7]	[−0.0291, −0.0149]	[−446.8, −216.7]
Quarter 2 ATT	−0.120***	−1639***	−0.0314***	−458.3***	−0.0212***	−307.6***
	(0.00333)	(42.73)	(0.00221)	(35.86)	(0.00447)	(75.38)
	[−0.126, −0.113]	[−1723, −1555]	[−0.0357, −0.0270]	[−528.6, −388.0]	[−0.0299, −0.0124]	[−455.3, −159.8]
Quarter 3 ATT	−0.00714***	−142.1***	−0.0282***	−428.2***	−0.0333***	−470.8***
	(0.00341)	(48.15)	(0.00439)	(69.63)	(0.00424)	(68.50)
	[−0.0138, −0.000455]	[−236.5, −47.76]	[−0.0368, −0.0196]	[−564.6, −291.7]	[−0.0416, −0.0250]	[−605.0, −336.5]
Quarter 4 ATT	−0.0177***	−284.1***	−0.0249***	−346.0***	−0.0223***	−346.7***
	(0.00394)	(57.70)	(0.00311)	(45.78)	(0.00320)	(60.16)
	[−0.0254, −0.00997]	[−397.2, −171.1]	[−0.0310, −0.0188]	[−435.7, −256.3]	[−0.0286, −0.0160]	[−464.6, −228.8]
Quarter 5 ATT	−0.0279***	−390.2***	−0.00505	−87.34	−0.0376***	−546.6***
	(0.00415)	(58.46)	(0.00462)	(64.40)	(0.00298)	(54.69)
	[−0.0361, −0.0198]	[−504.8, −275.7]	[−0.0141, 0.00401]	[−213.5, 38.88]	[−0.0434, −0.0317]	[−653.8, −439.4]
Quarter 6 ATT	−0.0218***	−341.1***	3.91e−05	−5.505	‐‐	‐‐
	(0.00514)	(72.40)	(0.00631)	(84.75)		
	[−0.0319, −0.0118]	[−483.0, −199.1]	[−0.0123, 0.0124]	[−171.6, 160.6]		
Quarter 7 ATT	−0.00560	−96.60	0.0118	131.0	‐‐	‐‐
	(0.00565)	(75.14)	(0.00839)	(111.1)		
	[−0.0167, 0.00547]	[−243.9, 50.67]	[−0.00464, 0.0282]	[−86.66, 348.7]		
Quarter 8 ATT	−0.0280***	−408.1***	‐‐	‐‐	‐‐	‐‐
	(0.00666)	(88.04)				
	[−0.0410, −0.0149]	[−580.7, −235.6]				

*Note*: Quarter‐specific ATTs estimated using Callaway and Sant’Anna (2021) estimator based on doubly robust estimation as specified in Callaway and Sant’Anna (2021) and Sant’Anna and Zhao (2020). Never treated units are control observations. Pre‐reform 2016 covariates in regression include county‐level unemployment rate, dentists per capita and real median household income in 2016 dollars. Cluster robust standard errors by state in parentheses. 95% confidence intervals in brackets. Twenty‐one states in each regression include the three treatment states (Indiana, Missouri and Nebraska) and 18 states which were fully fee‐for‐service during the study period.

Abbreviations: ATT, Average Treatment Effect on the Treated; FFS, fee‐for‐service.

****p* < 0.01; ***p* < 0.05; **p* < 0.1. Dates states transitioned to managed care are: Jan. 1, 2017 (Indiana); May 1, 2017 (Missouri); Oct. 1, 2017 (Nebraska).

*Source*: 2016–2018 T‐MSIS Medicaid and CHIP Analytic Claims Data from children ages 0–18. Unemployment rate: U.S. Bureau of Labor Statistics. Dentists per capita: American Dental Association. Median Household Income: United States Census.

**TABLE A4:** Aggregate Callaway and Sant’Anna (2021) ATT policy estimate of transition from FFS to dental managed care on dental care utilization

	Share of beneficiaries with a dental claim	Visits per 10,000 beneficiaries
Overall aggregate ATT policy estimate	−0.035***	−504.06***
	(0.0068)	(93.03)
	[−0.048, −0.022]	[−686.4, −321.7]

*Note*: Overall ATT estimated using Callaway and Sant’Anna (2021) estimator based on doubly robust estimation as specified in Callaway and Sant’Anna (2021) and Sant’Anna and Zhao (2020). Never treated units are control observations. Pre‐reform 2016 covariates in regression include county‐level unemployment rate, dentists per capita and real median household income in 2016 dollars. Standard errors in parentheses. 95% confidence intervals in brackets. Twenty‐one states in each regression include the three treatment states (Indiana, Missouri and Nebraska) and 18 states which were fully fee‐for‐service during the study period.

Abbreviations: ATT, Average Treatment Effect on the Treated; FFS, fee‐for‐service.

****p* < 0.01; ***p* < 0.05; **p* < 0.1. Dates states transitioned to managed care are: Jan. 1, 2017 (Indiana); May 1, 2017 (Missouri); Oct. 1, 2017 (Nebraska).

*Source*: 2016–2018 T‐MSIS Medicaid and CHIP Analytic Claims Data from children ages 0–18. Unemployment rate: U.S. Bureau of Labor Statistics. Dentists per capita: American Dental Association. Median Household Income: United States Census.

**TABLE A5:** ATTs. Policy effects of transition from FFS to dental managed care on share of beneficiaries with a dental diagnostic claim by quarter

	Indiana	Missouri	Nebraska
Aggregated ATT	−0.0402***	−0.0137	−0.009**
	(0.004)	(0.006)	(0.003)
	[−0.052, −0.027]	[−0.033, 0.005]	[−0.017, −0.0002]
Quarter 1 ATT	−0.100***	−0.0208***	−0.00861*
	(0.00231)	(0.00295)	(0.00279)
	[−0.108, −0.093]	[−0.031, −0.011]	[−0.017, 0.0002]
Quarter 2 ATT	−0.115***	−0.0307***	−0.00990**
	(0.00270)	(0.00268)	(0.00256)
	[−0.123, −0.107]	[−0.040, −0.022]	[−0.018, −0.003]
Quarter 3 ATT	−0.00881**	−0.0242***	−0.0122**
	(0.00256)	(0.00416)	(0.00338)
	[−0.016, −0.002]	[−0.040, −0.009]	[−0.023, −0.003]
Quarter 4 ATT	−0.0167***	−0.0237**	0.00344
	(0.00344)	(0.00475)	(0.00362)
	[−0.028, −0.006]	[−0.039, −0.008]	[−0.008, 0.013]
Quarter 5 ATT	−0.0272***	−0.00937	−0.0155**
	(0.00394)	(0.00748)	(0.00402)
	[−0.039, −0.014]	[−0.038, 0.021]	[−0.029, −0.003]
Quarter 6 ATT	−0.0233**	0.000138	‐‐
	(0.00572)	(0.00908)	
	[−0.043, −0.003]	[−0.038, 0.044]	
Quarter 7 ATT	−0.00553	0.0126	‐‐
	(0.00670)	(0.0110)	
	[−0.035, 0.021]	[−0.054, 0.091]	
Quarter 8 ATT	−0.0252	‐‐	‐‐
	(0.00784)		
	[−0.066, 0.022]		

*Note*: Quarter‐specific ATTs estimated by ETWFE using Equation (2). Cohort‐specific aggregated ATT estimated using Equation (4). Pre‐reform 2016 covariates in regression include county‐level unemployment rate, dentists per capita and real median household income in 2016 dollars. Standard errors in parentheses. For the aggregated ATT, the standard errors were estimated using the delta method. For the cohort and quarter‐specific ATTs, cluster robust standard errors were calculated. 95% confidence intervals in brackets. *p*‐values and 95% confidence intervals generated from wild bootstrap with 9999 replications. Twenty‐one states in each regression include the three treatment states (Indiana, Missouri and Nebraska) and 18 states which were fully fee‐for‐service during the study period.

Abbreviations: ATT, Average Treatment Effect on the Treated; ETWFE, Extended two‐way fixed effects; FFS, Fee‐for‐service.

****p* < 0.01; ***p* < 0.05; **p* < 0.1. Dates states transitioned to managed care are: Jan. 1, 2017 (Indiana); May 1, 2017 (Missouri); Oct. 1, 2017 (Nebraska). Number of observations: 13,032. Number of counties: 1086.

*Source*: 2016–2018 T‐MSIS Medicaid and CHIP Analytic Claims Data from children ages 0–18. Unemployment rate: U.S. Bureau of Labor Statistics. Dentists per capita: American Dental Association. Median Household Income: United States Census.

**TABLE A6:** ATTs. Policy effects of transition from FFS to dental managed care on share of beneficiaries with a dental preventive claim by quarter

	Indiana	Missouri	Nebraska
Aggregated ATT	−0.0380***	−0.0173*	−0.0119***
	(0.004)	(0.005)	(0.003)
	[−0.049, −0.025]	[−0.035, 0.001]	[−0.020, −0.004]
Quarter 1 ATT	−0.0950***	−0.0220***	−0.00650*
	(0.00229)	(0.00263)	(0.00275)
	[−0.103, −0.088]	[−0.032, −0.013]	[−0.015, 0.0009]
Quarter 2 ATT	−0.109***	−0.0322***	−0.0104***
	(0.00244)	(0.00264)	(0.00255)
	[−0.116, −0.102]	[−0.042, −0.024]	[−0.019, −0.004]
Quarter 3 ATT	−0.00747**	−0.0287***	−0.0196***
	(0.00262)	(0.00389)	(0.00325)
	[−0.016, −0.00006]	[−0.044, −0.015]	[−0.030, −0.011]
Quarter 4 ATT	−0.0155***	−0.0285***	−0.00446
	(0.00326)	(0.00452)	(0.00394)
	[−0.026, −0.006]	[−0.043, −0.014]	[−0.019, 0.006]
Quarter 5 ATT	−0.0257***	−0.0119	−0.0186***
	(0.00378)	(0.00705)	(0.00390)
	[−0.037, −0.013]	[−0.038, 0.014]	[−0.031, −0.008]
Quarter 6 ATT	−0.0223**	−0.00534	‐‐
	(0.00540)	(0.00858)	
	[−0.040, −0.005]	[−0.044, 0.036]	
Quarter 7 ATT	−0.00385	0.00742	‐‐
	(0.00644)	(0.0106)	
	[−0.033, 0.022]	[−0.058, 0.070]	
Quarter 8 ATT	−0.0250*	‐‐	‐‐
	(0.00754)		
	[−0.063, 0.015]		

*Note*: Quarter‐specific ATTs estimated by ETWFE using Equation (2). Cohort‐specific aggregated ATT estimated using Equation (4). Pre‐reform 2016 covariates in regression include county‐level unemployment rate, dentists per capita and real median household income in 2016 dollars. Standard errors in parentheses. For the aggregated ATT, the standard errors were estimated using the delta method. For the cohort and quarter‐specific ATTs, cluster robust standard errors were calculated. 95% confidence intervals in brackets. *p*‐values and 95% confidence intervals generated from wild bootstrap with 9999 replications. Twenty‐one states in each regression include the three treatment states (Indiana, Missouri and Nebraska) and 18 states which were fully fee‐for‐service during the study period.

Abbreviations: ATT, Average Treatment Effect on the Treated; ETWFE, Extended two‐way fixed effects; FFS, fee‐for‐service.

****p* < 0.01; ***p* < 0.05; **p* < 0.1. Dates states transitioned to managed care are: Jan. 1, 2017 (Indiana); May 1, 2017 (Missouri); Oct. 1, 2017 (Nebraska). Number of observations: 13,032. Number of counties: 1086.

*Source*: 2016–2018 T‐MSIS Medicaid and CHIP Analytic Claims Data from children ages 0–18. Unemployment rate: U.S. Bureau of Labor Statistics. Dentists per capita: American Dental Association. Median Household Income: United States Census.

**TABLE A7:** ATTs. Policy effects of transition from FFS to dental managed care on share of beneficiaries with a dental prophylaxis claim by quarter

	Indiana	Missouri	Nebraska
Aggregated ATT	−0.0363***	−0.013	−0.006
	(0.004)	(0.005)	(0.003)
	[−0.047, −0.024]	[−0.031, 0.006]	[−0.014, 0.003]
Quarter 1 ATT	−0.0908***	−0.0180***	−0.00877*
	(0.00223)	(0.00258)	(0.00253)
	[−0.098, −0.084]	[−0.028, −0.009]	[−0.018, 0.0007]
Quarter 2 ATT	−0.105***	−0.0264***	−0.00477
	(0.00237)	(0.00244)	(0.00277)
	[−0.112, −0.098]	[−0.036, −0.018]	[−0.014, 0.004]
Quarter 3 ATT	−0.00744**	−0.0241***	−0.00865*
	(0.00234)	(0.00400)	(0.00296)
	[−0.015, −0.0006]	[−0.042, −0.009]	[−0.019, 0.0007]
Quarter 4 ATT	−0.0154**	−0.0225**	0.00610
	(0.00340)	(0.00447)	(0.00340)
	[−0.028, −0.004]	[−0.038, −0.007]	[−0.006, 0.016]
Quarter 5 ATT	−0.0240***	−0.00793	−0.0127*
	(0.00377)	(0.00653)	(0.00357)
	[−0.036, −0.011]	[−0.036, 0.019]	[−0.026, 0.0002]
Quarter 6 ATT	−0.0210**	−0.000959	‐‐
	(0.00499)	(0.00784)	
	[−0.038, −0.004]	[−0.039, 0.037]	
Quarter 7 ATT	−0.00368	0.0119	‐‐
	(0.00577)	(0.00970)	
	[−0.029, 0.019]	[−0.075, 0.077]	
Quarter 8 ATT	−0.0233	‐‐	‐‐
	(0.00695)		
	[−0.066, 0.019]		

*Note*: Quarter‐specific ATTs estimated by ETWFE using Equation (2). Cohort‐specific aggregated ATT estimated using Equation (4). Pre‐reform 2016 covariates in regression include county‐level unemployment rate, dentists per capita and real median household income in 2016 dollars. ETWFE: Extended two‐way fixed effects. Standard errors in parentheses. For the aggregated ATT, the standard errors were estimated using the delta method. For the cohort and quarter‐specific ATTs, cluster robust standard errors were calculated. 95% confidence intervals in brackets. *p*‐values and 95% confidence intervals generated from wild bootstrap with 9999 replications. Twenty‐one states in each regression include the three treatment states (Indiana, Missouri and Nebraska) and 18 states which were fully fee‐for‐service during the study period.

Abbreviations: ATT, Average Treatment Effect on the Treated; FFS, Fee‐for‐service.

****p* < 0.01; ***p* < 0.05; **p* < 0.1. Dates states transitioned to managed care are: Jan. 1, 2017 (Indiana); May 1, 2017 (Missouri); Oct. 1, 2017 (Nebraska). Number of observations: 13,032. Number of counties: 1086.

*Source*: 2016–2018 T‐MSIS Medicaid and CHIP Analytic Claims Data from children ages 0–18. Unemployment rate: U.S. Bureau of Labor Statistics. Dentists per capita: American Dental Association. Median Household Income: United States Census.

**TABLE A8:** ATTs. Policy effects of transition from FFS to dental managed care on share of beneficiaries with a dental fluoridation claim by quarter

	Indiana	Missouri	Nebraska
Aggregated ATT	−0.0357***	−0.015*	−0.008**
	(0.004)	(0.005)	(0.002)
	[−0.046, −0.023]	[−0.032, 0.003]	[−0.016, −0.0009]
Quarter 1 ATT	−0.0889***	−0.0192***	−0.00420
	(0.00217)	(0.00239)	(0.00242)
	[−0.09619, −0.08221]	[−0.02799, −0.01116]	[−0.01184, 0.00224]
Quarter 2 ATT	−0.103***	−0.0288***	−0.00691**
	(0.00224)	(0.00246)	(0.00238)
	[−0.1095, −0.09657]	[−0.03743, −0.02061]	[−0.01528, −0.0002861]
Quarter 3 ATT	−0.00675*	−0.0255***	−0.0154***
	(0.00243)	(0.00360)	(0.00296)
	[−0.01443, 0.0001069]	[−0.03964, −0.01297]	[−0.02492, −0.0071]
Quarter 4 ATT	−0.0145***	−0.0262***	−0.00111
	(0.00298)	(0.00425)	(0.00385)
	[−0.02459, −0.005315]	[−0.04012, −0.01217]	[−0.01555, 0.01001]
Quarter 5 ATT	−0.0244***	−0.0103	−0.0135**
	(0.00356)	(0.00662)	(0.00354)
	[−0.03519, −0.01211]	[−0.03614, 0.01534]	[−0.025, −0.003756]
Quarter 6 ATT	−0.0214**	−0.00350	‐‐
	(0.00508)	(0.00816)	
	[−0.03815, −0.004499]	[−0.04239, 0.03584]	
Quarter 7 ATT	−0.00354	0.00874	‐‐
	(0.00613)	(0.00989)	
	[−0.03211, 0.02027]	[−0.05342, 0.06373]	
Quarter 8 ATT	−0.0232*	‐‐	‐‐
	(0.00704)		
	[−0.05911, 0.0142]		

*Note*: Quarter‐specific ATTs estimated by ETWFE using Equation (2). Cohort‐specific aggregated ATT estimated using Equation (4). Pre‐reform 2016 covariates in regression include county‐level unemployment rate, dentists per capita and real median household income in 2016 dollars. ETWFE: Extended two‐way fixed effects. Standard errors in parentheses. For the aggregated ATT, the standard errors were estimated using the delta method. For the cohort and quarter‐specific ATTs, cluster robust standard errors were calculated. 95% confidence intervals in brackets. *p*‐values and 95% confidence intervals generated from wild bootstrap with 9999 replications. Twenty‐one states in each regression include the three treatment states (Indiana, Missouri and Nebraska) and 18 states which were fully fee‐for‐service during the study period.

Abbreviations: ATT, Average Treatment Effect on the Treated; FFS, Fee‐for‐service.

****p* < 0.01; ***p* < 0.05; **p* < 0.1. Dates states transitioned to managed care are: Jan. 1, 2017 (Indiana); May 1, 2017 (Missouri); Oct. 1, 2017 (Nebraska). Number of observations: 13,032. Number of counties: 1086.

*Source*: 2016–2018 T‐MSIS Medicaid and CHIP Analytic Claims Data from children ages 0–18. Unemployment rate: U.S. Bureau of Labor Statistics. Dentists per capita: American Dental Association. Median Household Income: United States Census.

**TABLE A9:** ATTs. Policy effects of transition from FFS to dental managed care on share of beneficiaries with a dental restorative claim by quarter

	Indiana	Missouri	Nebraska
Aggregated ATT	−0.0108*	−0.005	−0.004
	(0.002)	(0.002)	(0.001)
	[−0.019, 0.002]	[−0.014, 0.005]	[−0.008, 0.001]
Quarter 1 ATT	−0.0264***	−0.00525	−0.00228
	(0.00162)	(0.00134)	(0.00133)
	[−0.036, −0.020]	[−0.012, 0.002]	[−0.008, 0.003]
Quarter 2 ATT	−0.0306**	−0.0103***	−0.00247
	(0.00173)	(0.00127)	(0.00141)
	[−0.040, −0.022]	[−0.016, −0.004]	[−0.009, 0.004]
Quarter 3 ATT	−0.00382	−0.00799*	−0.00389*
	(0.00170)	(0.00173)	(0.00148)
	[−0.015, 0.004]	[−0.016, 0.001]	[−0.011, 0.0007]
Quarter 4 ATT	−0.00523	−0.00562	−0.00324
	(0.00199)	(0.00193)	(0.00159)
	[−0.020, 0.004]	[−0.014, 0.006]	[−0.011, 0.003]
Quarter 5 ATT	−0.00701	−0.00317	−0.00595*
	(0.00210)	(0.00258)	(0.00160)
	[−0.026, 0.009]	[−0.016, 0.018]	[−0.012, 0.0014]
Quarter 6 ATT	−0.00550	−0.00152	‐‐
	(0.00240)	(0.00317)	
	[−0.053, 0.010]	[−0.021, 0.034]	
Quarter 7 ATT	−0.00142	0.00126	‐‐
	(0.00273)	(0.00377)	
	[−0.111, 0.038]	[−0.199, 0.203]	
Quarter 8 ATT	−0.00652	‐‐	‐‐
	(0.00311)		
	[−0.133, 0.076]		

*Note*: Quarter‐specific ATTs estimated by ETWFE using Equation (2). Cohort‐specific aggregated ATT estimated using Equation (4). Pre‐reform 2016 covariates in regression include county‐level unemployment rate, dentists per capita and real median household income in 2016 dollars. Standard errors in parentheses. For the aggregated ATT, the standard errors were estimated using the delta method. For the cohort and quarter‐specific ATTs, cluster robust standard errors were calculated. 95% confidence intervals in brackets. *p*‐values and 95% confidence intervals generated from wild bootstrap with 9999 replications. Twenty‐one states in each regression include the three treatment states (Indiana, Missouri and Nebraska) and 18 states which were fully fee‐for‐service during the study period.

Abbreviations: ATT, Average Treatment Effect on the Treated; ETWFE, extended two‐way fixed effects; FES, fee‐for‐service.

****p* < 0.01; ***p* < 0.05; **p* < 0.1. Dates states transitioned to managed care are: Jan. 1, 2017 (Indiana); May 1, 2017 (Missouri); Oct. 1, 2017 (Nebraska). Number of observations: 13,032. Number of counties: 1086.

*Source*: 2016–2018 T‐MSIS Medicaid and CHIP Analytic Claims Data from children ages 0–18. Unemployment rate: U.S. Bureau of Labor Statistics. Dentists per capita: American Dental Association. Median Household Income: United States Census.
